# Black carbon in urban soils: land use and climate drive variation at the surface

**DOI:** 10.1186/s13021-024-00255-3

**Published:** 2024-03-02

**Authors:** Molly Burke, Erika Marín-Spiotta, Alexandra G. Ponette-González

**Affiliations:** 1 Department of Geography and the Environment, University of North Texas, 1155 Union Circle #305279, Denton, TX 76203 USA; 2 Department of Geography, University of Wisconsin-Madison, 550 North Park Street, Madison, WI 53706 USA; 3 Present Address: Department of Geography, University of Utah, Salt Lake City, UT 84112 USA; 4 Present Address: Department of City and Metropolitan Planning, University of Utah, Salt Lake City, UT 84112 USA; 5 Present Address: Natural History Museum of Utah, University of Utah, Salt Lake City, UT 84108 USA

**Keywords:** Air pollution, Cities, Land cover, Nature-based solutions, Organic carbon, Pyrogenic carbon, Roads

## Abstract

**Background:**

Black carbon (BC) encompasses a range of carbonaceous materials––including soot, char, and charcoal––derived from the incomplete combustion of fossil fuels and biomass. Urban soils can become enriched in BC due to proximity to these combustion sources. We conducted a literature review of BC in urban soils globally and found 26 studies reporting BC and total organic carbon (TOC) content collected to a maximum of 578 cm depth in urban soils across 35 cities and 10 countries. We recorded data on city, climate, and land use/land cover characteristics to examine drivers of BC content and contribution to TOC in soil.

**Results:**

All studies were conducted in the northern hemisphere, with 68% of the data points collected in China and the United States. Surface samples (0–20 cm) accounted for 62% of samples in the dataset. Therefore, we focused our analysis on 0–10 cm and 10–20 cm depths. Urban soil BC content ranged from 0–124 mg/g (median = 3 mg/g) at 0–10 cm and from 0–53 mg/g (median = 2.8 mg/g) at 10–20 cm depth. The median proportional contribution of BC to TOC was 23% and 15% at 0–10 cm and 10–20 cm, respectively. Surface soils sampled in industrial land use and near roads had the highest BC contents and proportions, whereas samples from residential sites had among the lowest. Soil BC content decreased with mean annual soil temperature.

**Conclusions:**

Our review indicates that BC comprises a major fraction (nearly one quarter) of the TOC in urban surface soils, yet sampling bias towards the surface could hide the potential for BC storage at depth. Land use emerged as an importer driver of soil BC contents and proportions, whereas land cover effects remain uncertain. Warmer and wetter soils were found to have lower soil BC than cooler and drier soils, differences that likely reflect soil BC loss mechanisms. Additional research on urban soil BC at depth and from diverse climates is critical to better understand the role of cities in the global carbon cycle.

**Supplementary Information:**

The online version contains supplementary material available at 10.1186/s13021-024-00255-3.

## Introduction

Storage of soil organic carbon (SOC) is often proposed as a nature-based solution to climate change, with much focus on the potential for soil carbon (C) sequestration in managed lands, including agricultural areas, parks, and forests [1–3]. Recent research shows that SOC content in the surface soils of urban greenspaces is on par with that measured in adjacent natural ecosystems [4], underscoring the important role that urban soils may also play in C storage [1, 5–7].

Organic C stored in soils includes organic matter derived from plant, animal, and microbial organisms at different stages of decomposition and black carbon (BC), a byproduct of incomplete fossil fuel and biomass combustion. Yet, many of the studies that quantify urban soil C do not distinguish between these two fractions [4, 5, 8]. The contribution of BC to SOC can nevertheless be significant [9], with a recent global synthesis of BC in SOC across land use types, fire regimes, and soil types showing that BC can account for as much as 40% of SOC in urban soils [10].

Black carbon is a continuum of combustion products ranging from coarse particles of slightly charred biomass to highly condensed submicron-sized particles of soot and graphite [11, 12]. Although the physical and chemical properties of BC vary along this continuum, all forms of BC are high in C content, chemically heterogeneous, and have an aromatic core [11]. The aromaticity and associated low reactivity of BC particles cause it to have longer turnover times in soil than non-combusted organic C [12, 13]. In addition, BC particles sorb minerals and organic compounds, leading to the physical protection of BC from microbial decomposition [14, 15]. The chemical heterogeneity of BC can also make BC less biodegradable. In sum, BC is characterized by thermal and chemical stability and can persist in the soil profile longer than other forms of organic C [13, 16, 17]. Estimated residence times for BC range from hundreds to thousands of years [12].

Globally, biomass burning is the most important source of BC to the environment [14, 15]. However, in urban areas, BC is primarily emitted from diesel powered vehicles, stationary fuel combustion (e.g., coal), residential fuel combustion, and industrial processes [18]. After emission, particle size determines the distance that BC can travel before its deposition to soils in precipitation (wet deposition; [19] or in dry form [11, 18]. Smaller particles (e.g., soot) can reside in the atmosphere for days to weeks [20, 21], while larger particles fall out quickly and enter soils close to the source. Once incorporated into the soil, BC can accumulate in the soil profile or be lost via wind and water erosion [12, 22–24]. In the soil, BC is susceptible to breakdown by fungal hyphae [22, 24]. When exported or deposited to water bodies, BC can undergo photodegradation [25, 26].

Urban areas are biogeochemical hotspots [27], with high C emissions and high potential for C storage [28]. However, urban soil C storage is not currently well represented in C cycle models [28, 29]. It is therefore important to understand the role of cities in global C cycling by accounting for urban C sources and sinks. Accurate measurements of SOC components (BC and total organic carbon (TOC)) are needed to better understand urban C sinks, specifically, given the differential contribution of these fractions to long-term C storage.

To address this key knowledge gap, we compiled data from 26 global studies reporting measurements of urban soil BC contents and proportions. Here, we use the term “black carbon” instead of “pyrogenic carbon” given the prevalence of the term in the urban soil literature. We also examined land use/land cover and climatic variables (i.e., mean annual soil temperature, mean annual precipitation) as potential drivers of BC contents and contribution to urban soils.

## Methods

### Literature search and study selection

We assembled data on BC and TOC content in urban soils using a three-step process. Studies were identified, screened for eligibility, and then selected for inclusion in the dataset. We conducted a Web of Science literature search to identify all (i.e., no predetermined time frame) existing peer-reviewed studies published in English-language journals using the following search string: “urban soil*” AND “black carbon” OR “elemental carbon” OR “fire derived carbon” OR “pyrogenic carbon”. The initial search yielded 1973 studies. The titles and abstracts of all studies were screened to determine whether they contained data on BC content in urban soils. A site was classified as “urban” if the study categorized it as “urban”, or if the site was within a developed location with a population of at least 5000 residents [30]. A total of 33 sources, or approximately 2%, met the initial screening criteria. Most of the studies not selected for review reported BC in air and sediment, not soil, or were conducted in non-urban areas. References cited in the 33 studies (*n* = 2003) were screened using the same criteria to find other potential studies. Four additional relevant studies were identified as eligible for inclusion in the dataset. Finally, we identified one unpublished thesis that did not appear in the search [31].

All publications identified through the search process were subjected to full review before final selection. We removed studies from consideration if soil samples from urban and non-urban soils were composited, as this prevented us from determining urban soil BC content. We also excluded papers with unclear protocols for BC determination and papers that duplicated data reported in previous studies. In these cases, the earliest publication was included in the synthesis. In total, 26 studies were selected that fulfilled the above criteria (Additional file 1: Table S1).

### Data extraction and coding process

From these studies, we extracted data on BC and TOC content, and on city, climate, soil, land use and land cover characteristics, as well as on sampling and analytical protocols. Data were recorded at two scales: the city scale and the site scale.

For each city, we recorded information on latitude, longitude, population size, and elevation (m asl). If geographic coordinates were not provided for a city, we used Google Earth to obtain coordinate information as well as elevation data. In cases with two sources of elevation data (i.e., the study, Google Earth), we used the reported elevation in the publication. For population, we chose to use Google Earth data to keep census timeframe information consistent across studies.

To characterize the climate of each city, we obtained data on mean annual precipitation (mm/yr) and surface soil temperature (°C). For cities outside of the United States, we used precipitation data from the *United Nations World Meteorological Organization Standard Normals Dataset* [32]. In this dataset, precipitation amounts are reported as 30-year normals for 1961–1990. If a city was not listed in this dataset, we reported the value from the closest meteorological station. We found that 78.5% of all international cities in the studies were within 0–50 km of a meteorological station. For cities within the United States, we recorded data from the *U.S. Climate Normals 2020: U.S. Annual/Seasonal Climate Normals (1961–1990) dataset* provided by NOAA’s National Centers for Environmental Information Service [33]. For consistency with the international cities in our dataset, we calculated the mean annual precipitation normals for the years 1961–1990. Five of the study cities (Cleveland, OH, Denton, TX, Detroit, MI, Tacoma, WA, and San Juan, PR) had gaps in the available data record. In these cities, we took the mean of 21, 29, 7, 28, and 27 years, respectively, from 1961–1990. Annual mean soil temperature data were obtained from the *Global Soil Bioclimatic variables at 30 arc second resolution* dataset [34]. This dataset includes soil temperature at 0–5 cm and 5–15 cm depth. When reported, we also recorded soil characteristics such as soil order, pH, and clay content.

We recorded intraurban land use at each of the sampling sites when this information was provided by the authors. Otherwise, we contacted the authors for additional information. For sites within the United States, we used the *National Land Cover Database* and interactive map provided by the *Multi Resolution Land Characterization Consortium* (MRLC) to determine land use [35]. We were unable to determine land use at 16% of the sites in our dataset because specific sampling site coordinates were not provided.

Land use was divided into nine categories (Table 1). Agricultural land encompasses urban gardens and farmlands. Recreational land use includes parks, sport fields, and urban trails. Residential areas are lands used predominantly for housing. Low-intensity land use applies to urban areas with little developed land but that still receive urban amenities such as water, electricity, sewage, and emergency services. Medium-intensity land use applies to urbanized areas with a mixture of land uses in development, such as urban transit, housing, retail services and commercial development. High-intensity land uses are densely constructed areas with compact residential living, commercial institutions, and intermixing of land uses and development. We defined commercial land use as areas used to generate profit such as storefronts, warehouses, and office buildings. Industrial land use includes manufacturing plants, mining facilities, and areas used for generating public utilities. Transportation land use includes areas such as roadways, roadsides, and railways which experience high levels of traffic.

**Table 1:** Land use categories used to characterize soil sampling sites included in this review of black carbon (BC) in urban soils

land use	Definition	References
Agricultural	Urban gardens and farmlands	Author determination
Recreational	Parks, sport fields, and urban trails	Modified from [36]
Residential	Predominantly housing	Modified from [36]
Low Intensity	Urban areas with little developed land	Modified from NLCD: National Land Cover Database Class Legend and Description [35]
Medium Intensity	Fully urbanized areas with a mixture of land uses	Modified from NLCD: National Land Cover Database Class Legend and Description [35]
High Intensity	Densely constructed urban areas with compacted development	Modified from NLCD: National Land Cover Database Class Legend and Description [35]
Commercial	Areas used to generate profit such as storefronts, warehouses, and office buildings	Modified from [36]
Industrial	Manufacturing plants, and mining facilities	Modified from [36]
Transportation	Roadsides and railways that experience high levels of traffic	Modified from [36]

We assigned sampling sites to one of five land cover categories, which were based on physical study site descriptions (Table 2). Herbaceous cover includes sites with descriptions of grass patches, residential lawns, open fields, and sports fields. Samples collected from underneath tree cover were grouped into the category “tree”. Garden land cover includes lands used for urban crops and intentional planting. Roadside land cover includes sites near or at the edge of roads. Railway land cover was used to describe sites near a railway or train station. We were unable to assign a land cover category to ~ 50% of the sampling sites in our dataset.

**Table 2:** Land cover categories used to characterize soil sampling sites included in this review of black carbon (BC) in urban soils

Land cover	Definition	References
Herbaceous	Grass patches, lawns, open fields, and sports fields	Modified from NLCD: National Land Cover Database Class Legend and Description [35]
Garden	Urban crops and intentional planting	Author determination
Tree	Underneath tree cover	Author determination
Roadside	Edge of road or grassy strip	Modified from [37]
Railway	Railway or train station	Author determination

We also recorded the method of BC measurement used in each study. Several methods exist for measuring BC in soils and sediments, and each method detects a different portion of the BC combustion continuum [38]. Some thermal methods (e.g., chemo-thermal oxidation) detect the most refractory fractions of BC (e.g., soot, graphite), while other thermal (e.g., thermal-optical reflectance) and chemical (e.g., dichromate oxidation) methods are thought to measure a larger region of the combustion continuum [11, 38]. We also recorded the sampling depth. If sampling depths were included in a figure, but the values were not listed in the text or the supporting information, we used a Web Plot Digitizer to determine the value.

### Calculations and data analysis

Soil BC and TOC content were recorded in the units reported in the original publication and then converted to mg C/g of soil. As bulk density data were not reported for most studies in our dataset, we were unable to calculate BC stocks. Instead, we focused our analysis on BC and TOC content and the proportional contribution of BC to TOC.

We used descriptive statistics to examine the mean, median, and range of values across all samples and at 0–10 and 10–20 cm depths. We also evaluated variations in BC, TOC, and BC/TOC (%) by depth range. Data for deeper soil increments were limited to a subset of cities, and there was substantial variability among cities in the number of samples collected per depth range. For this reason, values were averaged for each city by depth range and compared using a non-parametric Kruskal–Wallis test followed by Steel–Dwass multiple comparisons.

Samples from 0–10 cm and 10–20 cm depth comprised 62% of all soil samples in our dataset, and thus we focused most of our analysis on these two depth ranges. Within these depth ranges, soil samples were collected to varying depths across studies, cities, and sampling sites. To account for this, we normalized BC content by dividing by the sample depth. We examined differences in BC contents and BC proportions among land use and land cover categories using a non-parametric Kruskal–Wallis test. Non-parametric Spearman’s rank correlations were used to examine relationships between BC contents and proportions and climatic variables because the data were non-normally distributed. Analyses were performed using JMPv14 (SAS institute INC). Statistical significance was set at *p* < 0.05.

## Results

Published between 2004 and 2023, the 26 studies reviewed here sampled urban soils across 35 cities and 10 countries. All cities are in the northern hemisphere, with 68% of the data points collected in China (45%) and the United States (23%). City population sizes range from ~ 66,000 to ~ 26,000,000 residents. Annual precipitation levels range from 202 mm/yr to 1780 mm/yr. All site locations are less than 500 m above sea level apart from one study conducted in Nepal (2200 m asl).

**Fig. 1:**
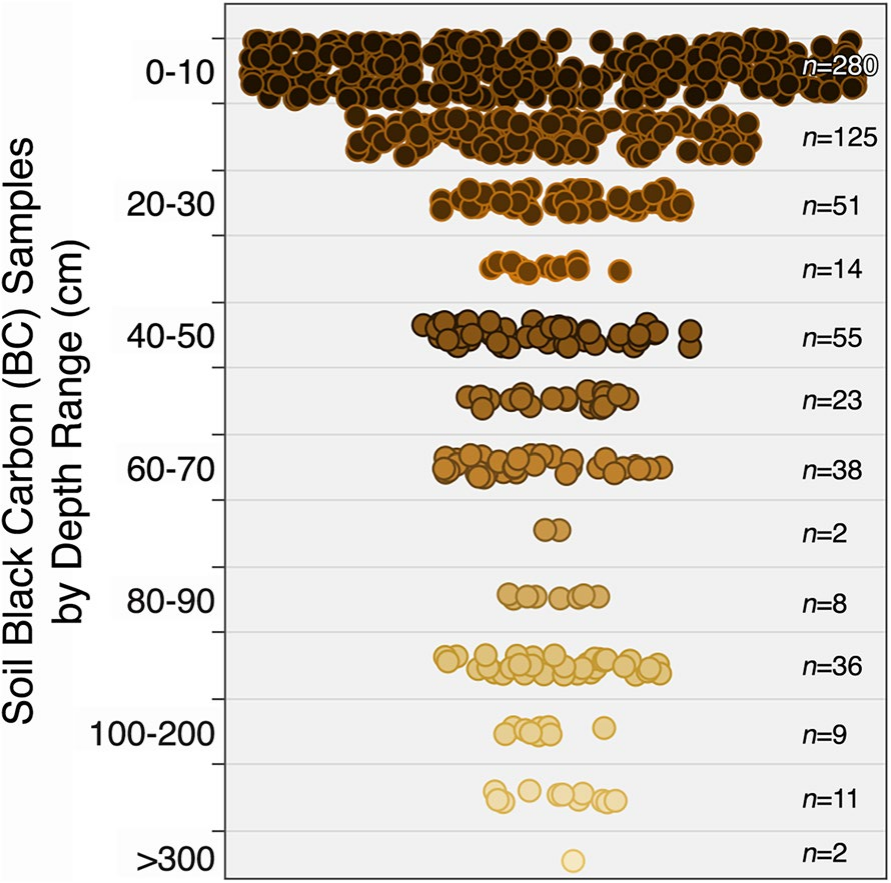
Distribution of soil black carbon (BC) samples included in this literature review grouped by sampling depth range. Each point represents one soil sample from the dataset. Soil depth is indicated by the color gradient (dark = closer to the surface, light = deeper in the soil). Spread between samples is meant to improve visualization. There is no x-axis

Soil samples were collected to a maximum 578 cm depth (Fig. 1), however most studies (84%) and cities (65%) within our dataset limited their BC data collection to the top 20 cm. Specifically, a total of 280 samples (43%) were taken from 0–10 cm depth, and 125 samples (19%) were taken from 10–20 cm depth. Approximately 40% of studies analyzed samples at more than one depth range. Only 2.5% of samples were collected to 1-m depth or below.

### Urban soil BC and TOC contents and BC proportions

**Fig. 2:**
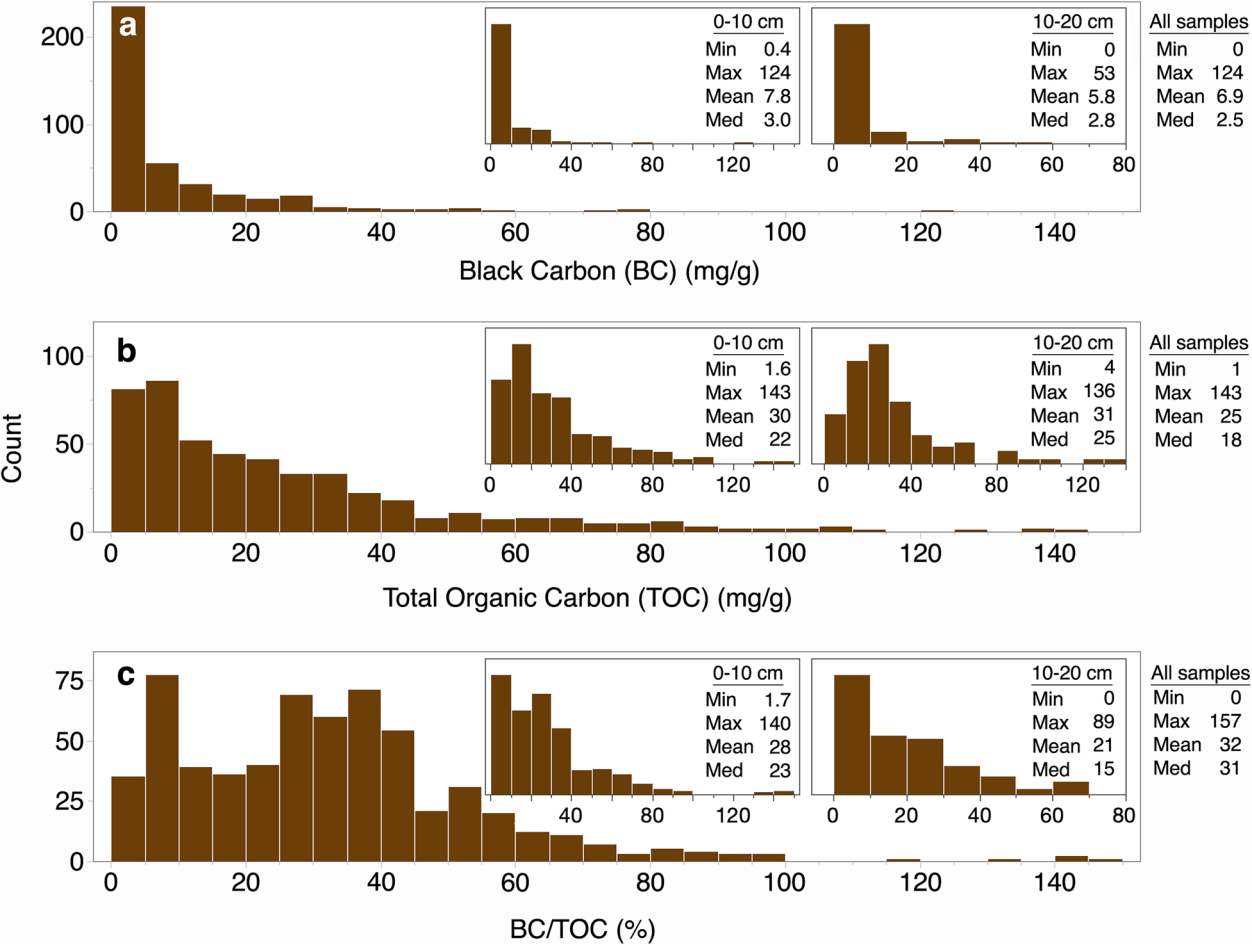
Histograms of soil black carbon (BC), total organic carbon (TOC) content (bins are 5 mg/g), and the contribution of BC to TOC (bins are 5 percent) across all sample depths, 0–10 cm depth (inset), and 10–20 cm depth (inset). Summary statistics include minimum, maximum, mean, and median. Note difference in y-axis scales

Across all samples and sample depths, soil BC ranged from 0–124 mg/g, with a median value of 2.5 mg/g (Fig. 2). Only ~ 10% of samples had contents > 20 mg/g. Soil TOC contents also ranged widely (1–143 mg/g), with a median of 18 mg/g. The contribution of BC to TOC ranged from 0 to 157% with a median value of ~ 31%.

While we did not detect significant differences in soil BC content with depth, TOC content was significantly higher at 0–10 cm (p = 0.001) and 10–20 cm (p = 0.03) compared to 60–70 cm depth. Conversely, the proportion of BC was marginally lower at 0–10 cm (p = 0.09) and significantly lower at 10–20 cm (p = 0.05) compared to 60–70 cm depth.

All studies used thermal or chemical measurement techniques: 62% of studies used a chemo-thermal oxidation (CTO) method, while 19% of studies used chemical oxidation with dichromate to quantify soil BC. Only 2 and 3 studies employed thermal-optical reflectance (TOT/R) and thermogravimetric analysis, respectively. There were too few data points to conduct a statistical comparison of BC measured using different methods, but overall, the dichromate oxidation method appeared to measure more BC/TOC than the thermal methods. At 0–10 cm, median BC/TOC was 12% (range 6–137%) in cities (*n* = 10) where the CTO method was used. The median BC/TOC value reported for the TOT/R method (18%, *n* = 4 cities) was similar to that of CTO, but the range of values was narrower (BC/TOC 17–26%). The chemical method (*n* = 2 cities) measured higher minimum (41%) and median (57%) soil BC/TOC compared to the other two thermal methods. At 10–20 cm depth, CTO was used in nine cities and dichromate oxidation in two cities. Where soil BC was measured using chemical oxidation, minimum (40%), median (54%), and maximum (69%) BC/TOC values were higher compared to the CTO values by fivefold, threefold, and 1.5-fold, respectively.

### Land use differences in urban soil carbon

**Fig. 3:**
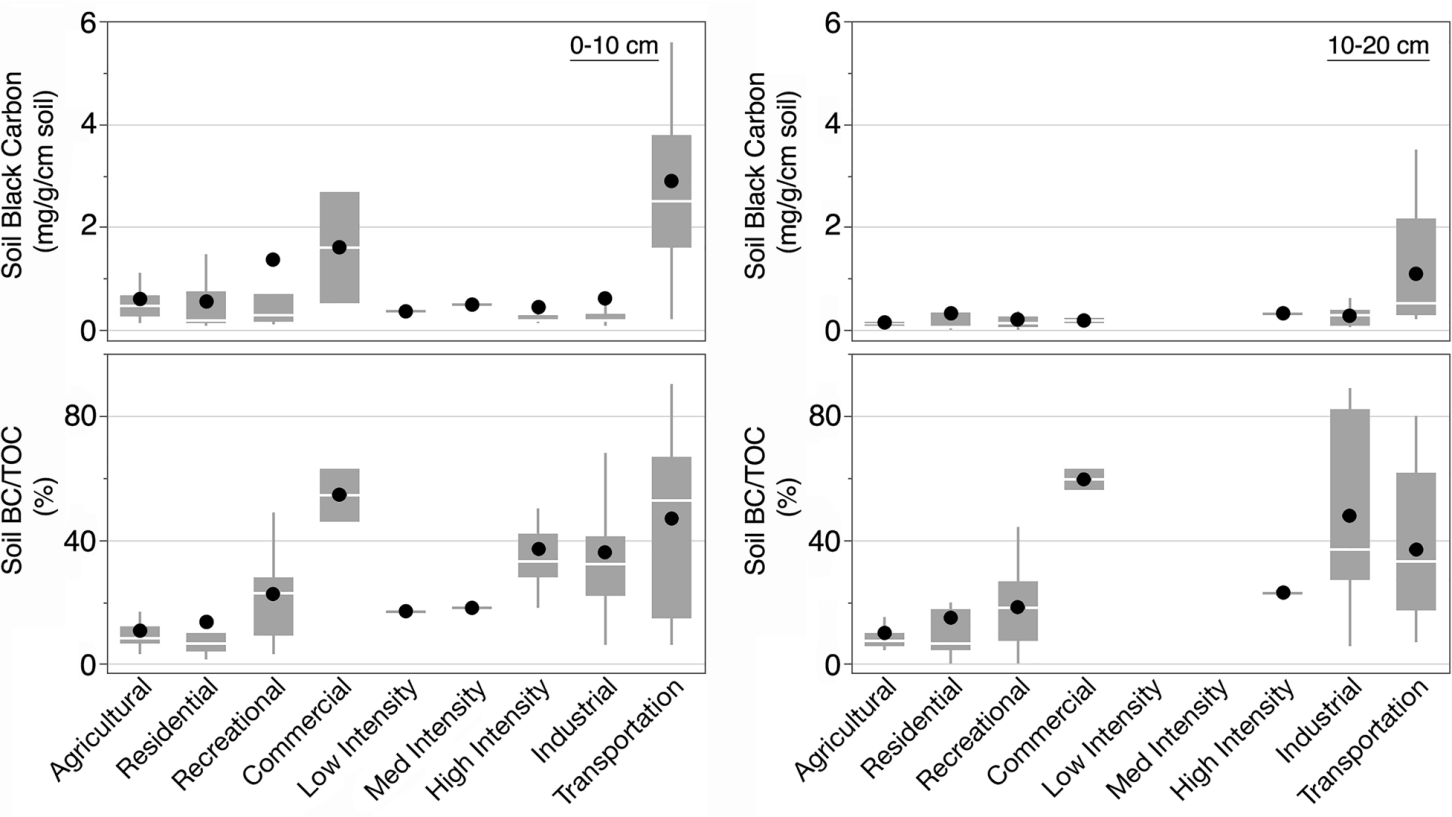
Black carbon (BC) content (mg/g) and the contribution of BC to TOC in urban soils as a function of land use at 0–10 cm and 10–20 cm depth. Dots indicate mean values. Missing bars are due to lack of land use data

Median soil BC content varied among land use categories at both 0–10 cm and 10–20 cm depths (Fig. 3). At 0–10 cm depth, median soil BC contents were highest (2.5 mg/g) and most variable (0.2–12.4 mg/g) in the transportation land use category. These were significantly greater compared to contents measured at industrial (0.18 mg/g), residential (0.19 mg/g), recreational (0.28 mg/g), and agricultural (0.46 mg/g) sites by approximately 14-fold, 13-fold, ninefold, and fivefold respectively. High intensity (0.19 mg/g) and industrial sites were significantly lower than urban agricultural sites by approximately twofold and threefold. Although industrial land use had the lowest median value, average BC content did not differ from residential and high-intensity land uses.

At 10–20 cm depth, median soil BC contents ranged from 0.07–0.53 mg/g. Similar to the results for soils at 0–10 cm, median soil BC contents were highest at transportation sites (0.53 mg/g) and lower at residential sites (0.07 mg/g). Median values for transportation sites were greater compared to residential, agricultural (0.12 mg/g), and recreational (0.13 mg/g) sites. There were no significant differences in soil BC contents among the other land use categories. However, in contrast to samples from 0–10 cm, high-intensity and industrial land uses had soil BC contents at the higher end of the range, whereas agricultural sites had values at the lower end of the observed range.

The contribution of BC to TOC was greater under intensive land use as well. At both 0–10 cm and 10–20 cm depths, median contribution of BC to TOC at industrial and commercial sites was higher than at residential, recreational, and agricultural sites. Transportation sites had greater proportions of BC than residential and recreational sites. Although sites used for recreation are generally considered low-intensity land use, the contribution of BC to TOC was significantly higher than at agricultural and residential sites at both depths.

### Land cover differences in urban soil carbon

Soil BC contents and proportions varied as a function of land cover within broad land use categories. At 0–10 cm soil depth, roadside sites (i.e., transportation land use) had median BC contents that were higher than at 10–20 cm depth. However, roadside sites had high proportions of BC at both depths. Railway sites at 0–10 cm depth had low BC contents, but high proportions of BC.

**Fig. 4:**
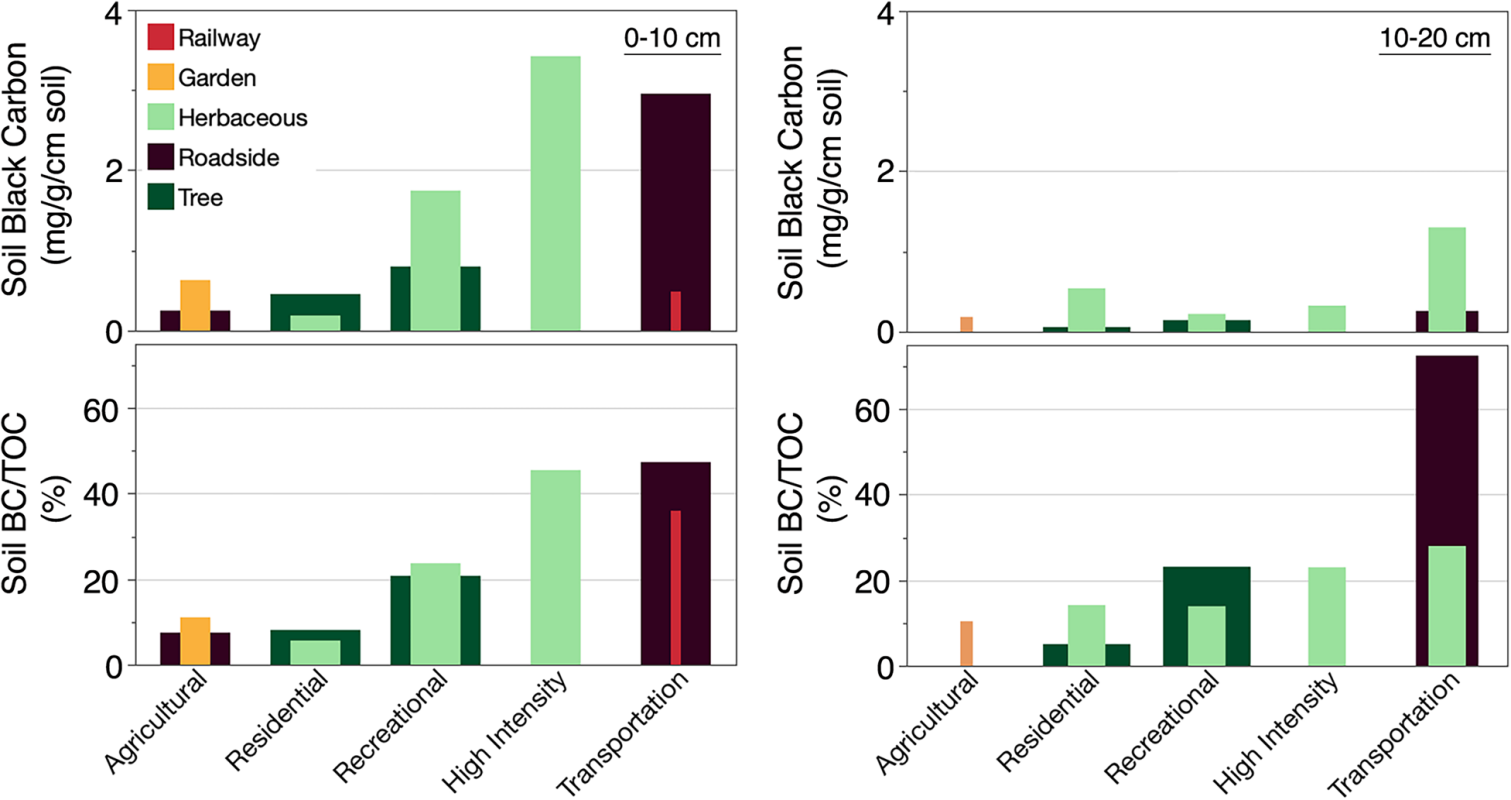
Mean black carbon (BC) contents (mg/g) and the contribution of BC to TOC in urban soils as a function of land use and land cover at 0–10 cm and 10–20 cm depth. Missing bars are due to lack of land use data

At 0–10 cm depth, the median content of soil BC sampled at recreational sites under herbaceous land cover was higher than under tree cover. At residential sites the opposite was true: soil BC was higher under tree cover than herbaceous land cover. Conversely, at 10–20 cm depth, soil BC sampled under herbaceous land cover was higher than tree cover in both residential and recreational land uses (Fig. 4).

### Correlations between soil carbon and climatic variables

At 0–10 cm and 10–20 cm depth, urban soil BC contents were negatively correlated with soil temperature (0–10 cm, ρ = − 0.38; 10–20 cm, ρ = − 0.56), and this relationship was stronger in deeper soils. At 0–10 cm depth, soil BC and TOC decreased with increasing soil temperature at similar rates. As a result, the contribution of BC to TOC did not change with soil temperature. At 10–20 cm depth, however, TOC did not change with soil temperature, which resulted in decreasing BC proportions with increasing soil temperature.

**Fig. 5:**
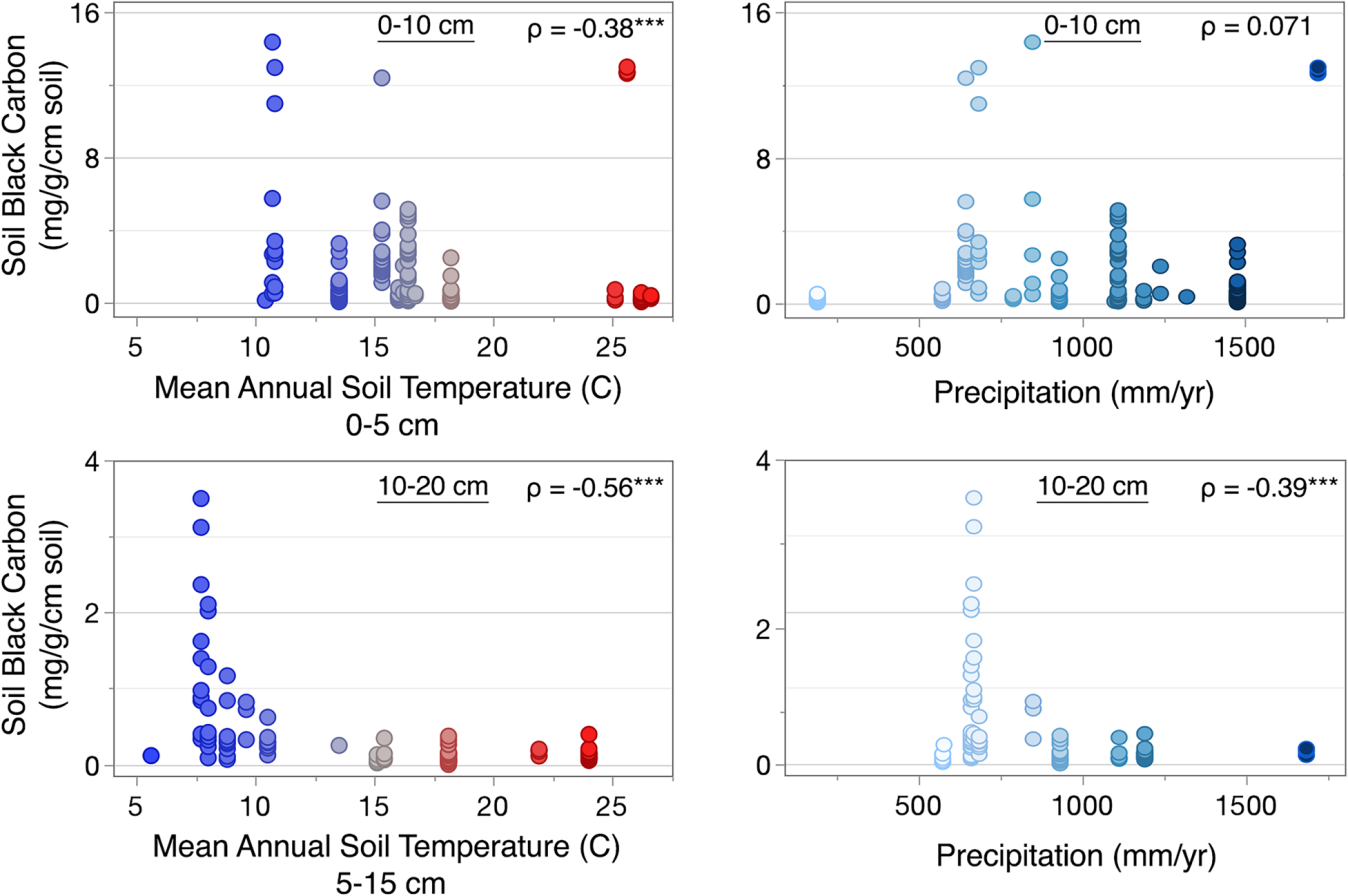
Non-parametric correlations between soil black carbon (BC) and mean annual soil temperature (left) and mean annual precipitation (right) in urban soils at 0–10 cm and 10–20 cm depths. *p < 0.05, **p < 0.01, ***p < 0.001

Urban soil BC contents were negatively correlated with annual precipitation at 10–20 cm depth (ρ = − 0.39). Soil BC and BC proportions were also negatively correlated with elevation, but only at 10–20 cm depth (ρ = − 0.38) (Fig. 5).

## Discussion

Although urban areas cover less than 5% of the land surface [39], they have the potential to store considerable amounts of C, including both organic C [6–8, 40] and BC [41–44]. In fact, our synthesis shows that, on average, BC comprises close to 25% of the TOC in surface soils (0–10 cm). By contrast, Reisser et al. [10] found that BC accounts for an average of ~ 10% of TOC in urban soils. Their 2016 synthesis included a much smaller number of studies, which could explain why they reported a lower proportion of BC. Of 55 articles, only two publications, one from 2010 and one from 2011, contained urban soil data. As our review highlights, in the past decade there has been a substantial increase in studies aimed at investigating urban soil [10]. It is therefore not surprising that most (82%) of the observations in our urban-focused dataset were published between 2012 and 2021, subsequent to those included in Reisser’s study.

Regardless of publication year, it appears that most of the work on soil BC focuses on the most biologically active portion of the soil, which is also the most superficial. In our dataset, sample depths ranged from 2–578 cm. Yet, samples from 0–10 cm accounted for approximately 43%, while samples from 0–20 cm accounted for 62% of all samples. Similarly, a recent review focused on soil-derived ecosystem services in urban ecosystems noted that 63% of the studies limited sampling depth to 20 cm [45], with only 12% of the studies analyzing samples down to 100 cm depth. Yost and Hartemink [46] examined soil depths reported in studies published in four soil science journals. Their analysis revealed that soil depths ranged from 2–560 cm, with an average sample depth of 23 cm, indicating the depth bias extends beyond just urban soils.

While our analysis focused on BC in surface soils, soils can store large amounts of C at depth (deep C here defined as > 30 cm [47]). The limited observations from deep soils in our dataset indicate that the contribution of BC to TOC peaks at intermediate depths (60–70 cm). Edmondson et al. [9] examined urban soil profiles in parks located in historically industrial areas in England and also found that the proportional contribution of BC to TOC increased with soil depth. They estimated that BC comprised 28–39% of the TOC storage to 1 m depth. The increasing proportion of BC with depth is likely due to preferential decomposition of less resistant C and vertical transport of BC from surface soils to deeper soils. Thus, the sampling depth bias towards surface soils could hide the potential for long-term (e.g., centuries to millennia; [12, 48] BC storage at depth.

### Land use drives variation in urban soil BC

We were not surprised to find that soil BC accumulation was higher in areas under high land use intensity and near known BC sources, such as transportation and industrial land use [49]. It is well documented that soil BC accumulates near roadsides where vehicle emissions are elevated [44, 50–54]. Furthermore, traffic emissions are the largest source of BC in urban areas [18]. Spatial patterns of soil contamination are also the product of current [10, 55, 56] and historical industrial processes [9, 42, 57]. For example, a study conducted in Nanjing, China, examining the vertical distribution of soil BC found greater BC content in samples from historical industrial and commercial sites than former residential sites [43]. Ongoing anthropogenic activities can increase exposure to BC sources, and past land uses can help explain the preservation of BC within the soil profile.

In our synthesis, soils sampled from urban agricultural land use contained the third highest BC contents after soils from transportation and industrial land use. In our review, the urban agricultural sites for which BC values are reported are located in China, Cuba, Germany, and the United States [55, 58–60], and the samples were largely collected in urban gardens. Gardens often contain high levels of organic matter, nutrients, and contaminants in topsoil [60–62]. It is well documented that BC is persistent in the soil profile [12, 63, 64] and that contamination from previous land use, such as agricultural biomass burning or industrial land use [42, 65], is likely still present in soils overturned for urban agricultural usage. Additionally, urban gardens located near sources of contamination such as roadways and industrial sites experience high levels of contamination from human activities [66]. Conversely, studies generally reported low BC contents in residential areas. This could be due to lower exposure to heavy traffic, industrial processes, and combustion sources [53, 67].

Land use impacts BC content as well as the proportional contribution of BC to TOC by altering the amount of BC and organic C [22]. For example, proximity to combustion sources coupled with loss of soil C as a result of disturbance (e.g., vegetation removal), soil capping, sealing, compaction, and construction would explain higher contributions of BC to TOC at transportation, industrial, and commercial sites [60, 68, 69]. By comparison, the lower contribution of BC to TOC at residential and agricultural sites is likely due to aboveground and belowground organic C inputs from vegetation and fewer BC sources [44]. Although we expected to also find lower BC proportions in recreational land uses (defined here as parks, athletic fields, and urban trails), this was not the case. Highly managed recreational landscapes often rely on maintenance practices, such as removal of clippings and leaf litter, that can deplete organic C inputs to soil [70], and in turn result in the proportional enrichment of BC.

### Uncertain land cover effects on urban soil BC

The effects of land cover on urban soil BC remain unclear. Previous studies show that urban trees capture and deliver more BC to the ground via leaf litterfall and throughfall than open grassy areas [71, 72]. Further, a study conducted in England found that tree-covered topsoil contained higher proportions of BC relative to TOC than grasslands [9]. Thus, we expected soil BC contents or proportions to be higher under tree cover than under herbaceous land cover. However, in residential and recreational land use, where soils were sampled beneath both tree and herbaceous land cover, we did not consistently find this pattern. One possible explanation for this is that observations from paired and unpaired sites were included in our dataset. As such, soils in some herbaceous sites may have been more exposed to BC emissions than those with tree cover. For example, at 10–20 cm depth, samples from herbaceous land cover had fivefold higher soil BC contents than samples from roadsides. However, these herbaceous samples were collected from grass verges, which are defined as vegetation strips located between sidewalks and roadways [37]. In other words, the herbaceous samples were likely exposed to high levels of emissions from gasoline-powered lawn and garden equipment [73] or vehicles. Very few of the studies in our dataset included specific land cover information. We need more data before we can state conclusive land cover effects on soil BC concentrations in heterogeneous landscapes. Differences in sampling strategy among studies and lack of detailed information on management effects within land uses and land covers can also contribute to high variability in concentrations of TOC and BC in urban soils.

### Urban soil BC is lower in warmer and wetter soils

Our study indicates that BC content tends to decline with increasing soil temperature and annual precipitation. This could be possibly due to increased microbial decomposition and loss via hydrologic flow paths. Black carbon decomposes via several processes, including chemical degradation (e.g., microbial decomposition) and can be lost from soils via wind and water erosion [22, 74]. Microbial decomposition is the most studied mechanism of BC loss and requires the presence of microorganisms capable of breaking down aromatic C compounds [22, 25, 75]. Typically, warmer soil temperatures increase microbial activity, accelerating decomposition [76]. Moisture also stimulates decomposition in soils [76, 77]. In urban areas, human activities affect air and surface temperatures (e.g., construction of impervious surfaces) and soil water content (e.g., irrigation), potentially contributing to accelerated levels of decomposition [39].

Black carbon can also be physically removed from soils through hydrologic transport and structural degradation of BC. Mechanisms such as shrink-swell [78, 79] and the vertical movement of water through soil systems redistributes surface BC to deeper in the profile [24]. While percolating water accounts for a small fraction of C mobilization, large amounts of BC could be lost due to surface runoff during rain events, especially in urban areas where a large proportion of the landscape surface is covered by impervious surfaces [24]. Evidence of BC particles in river sediment indicates that BC can effectively move into water systems via lateral erosion events [80]. Additionally, water can increase solubilization and hydrophobic properties of BC, increasing the leaching process [24, 74].

## Conclusions

Urban soils have the potential to store considerable amounts of BC. Our study synthesized published measurements to examine patterns and drivers of BC contents and proportions in urban soils. Our review indicates that BC comprises a significant fraction of the TOC in urban surface soils, yet sampling bias towards surface soils could hide the potential for BC storage at depth. Land use emerged as an importer driver of soil BC contents and proportions, whereas land cover effects remain uncertain. Warmer and wetter soils were found to have lower soil BC than cooler and drier soils, differences that likely reflect soil BC loss mechanisms. Taken together, our findings underscore the need to better understand the role of BC in the urban C cycle.

Urban soil C storage is not currently well represented in C cycle modeling, and it is therefore important to understand the role of cities by accounting for urban C sinks and sources. To improve our global understanding of BC and the C cycle, future research should consider sampling urban soils from deeper, more diverse climates. BC will continue accumulating in urban soils due to industrialization, vehicle emissions, and biomass burning, thus more global observations are required to better understand the role of soil BC in the urban C cycle.

### Supplementary Information

**Additional file 1. **Table of studies included in the literature review.

## Data Availability

The dataset generated for this study will be posted on the Knowledge Network for Biocomplexity (https://knb.ecoinformatics.org). The precipitation data for cities outside of the United States is available online at https://data.un.org/Data.aspx?d=CLINO&f=ElementCode%3A15. The precipitation data for cities within the United States is available online at https://www.ncei.noaa.gov/products/land-based-station/us-climate-normals. The soil temperature data is available online at https://zenodo.org/record/4558732#.ZFWEQi-B3yw. The land cover data used for cities within the United States is available online at https://www.mrlc.gov/viewer/.

## Abbreviations

BC: Black carbon
TOC: Total organic carbon
C: Carbon
SOC: Soil organic carbon
